# Evaluating Nationwide Application of Minimally Invasive Surgery for Treatment of Small Bowel Neuroendocrine Neoplasms

**DOI:** 10.1007/s00268-021-06036-0

**Published:** 2021-03-30

**Authors:** Enes Kaçmaz, Heinz-Josef Klümpen, Willem A. Bemelman, Els J. M. Nieveen van Dijkum, Anton F. Engelsman, Pieter J. Tanis

**Affiliations:** 1 Department of Surgery, Amsterdam UMC, University of Amsterdam, Cancer Center Amsterdam, Amsterdam, The Netherlands; 2 Cancer Center Amsterdam, Amsterdam, The Netherlands; 3 Department of Medical Oncology, Amsterdam UMC, University of Amsterdam, Amsterdam, The Netherlands; 4 Amsterdam UMC, ENETS Center of Excellence, University of Amsterdam, Amsterdam, The Netherlands; 5 Department of Surgery, Amsterdam UMC, Vrije Universiteit Amsterdam, Amsterdam, The Netherlands

## Abstract

**Aim:**

Open resection of small bowel neuroendocrine neoplasms (SB-NEN) is still considered standard-of-care, mainly because of frequently encountered multifocality and central mesenteric masses. The aim of this study was to evaluate surgical approach for SB-NEN at a national level and determine predictors for overall survival.

**Methods:**

Patients with SB-NEN who underwent resection between 2005 and 2015 were included from the Netherlands Cancer Registry. Patient and tumor characteristics were compared between laparoscopic and open approach. Overall survival was assessed by Kaplan–Meier and compared with the Log-rank test. Independent predictors were determined by Cox proportional hazards model.

**Results:**

In total, 482 patients were included, of whom 342 (71%) underwent open and 140 (29%) laparoscopic resection. The open resection group had significantly more multifocal tumors resected (24% vs. 14%), pN2 lymph nodes (18% vs. 7%) and stage IV disease (36% vs. 22%). Overall survival after open resection was significantly shorter compared to laparoscopic resection (3-year: 81% vs. 89%, 5-year: 71% vs. 84%, *p* = 0.004). In multivariable analysis, age above 60-years (60–75, HR 3.38 (95% CI 1.84–6.23); > 75 years, HR 7.63 (95% CI 3.86–15.07)), stage IV disease (HR 1.86 (95% CI 1.18–2.94)) and a laparoscopic approach (HR 0.51 (95% CI 0.28–0.94)) were independently associated with overall survival, whereas multifocal primary tumor, grade and resection margin status were not.

**Conclusion:**

Laparoscopic resection was the approach in 29% of SB-NEN at a national level with selection of the more favorable patients. Laparoscopic resection remained independently associated with better overall survival besides age and stage, but residual confounding cannot be excluded.

## Introduction

Small bowel neuroendocrine neoplasms (SB-NEN) are a rare type of gastrointestinal cancer and constitute 15% of all neoplasms of the jejunum and approximately 60% of the ileum, making it the most common gastroenteropancreatic NEN [1, 2]. Patients with stage I-III disease are amenable for curative resection, as well as selected stage IV patients with liver metastases [3, 4]. Resection remains the main treatment modality for these patients, resulting in relatively high 5-year overall survival rates of 70–80% for stage I-III and 35–80% for stage IV disease [3].

The majority of patients with SB-NEN already present with mesenteric lymph node metastases, and multifocal primary tumors can be found in up to 25–44% [5]. These disease characteristics make SB-NEN resection challenging. Although minimally invasive surgery is increasingly gaining acceptance as a standard approach for other gastrointestinal malignancies, minimally invasive surgery is still thought to potentially compromise oncological safety in SB-NEN, thereby potentially worsening survival outcomes [5]. Because of this, guidelines advise laparoscopic resection only in patients in which an appropriate intraoperative assessment of the bowel with proper segmental resection and adequate lymphadenectomy can be performed [3, 5].

Considering the evolution in the application of advanced laparoscopic resection for more complex oncological disease, and more specifically the experience with D3 mesenteric lymphadenectomy [6], application of minimally invasive surgery in SB-NEN might have increased as well. However, there are no population based data or prospective studies on this topic.

The primary aim of this study was to evaluate surgical approach for SB-NEN at a national level considering selection based on patient and tumor characteristics. Secondarily, the aim was to identify independent predictors of overall survival.

## Methods

### Study design

Data from all patients with SB-NEN diagnosed between 2010 and 2015 were extracted from the Netherlands Cancer Registry (NCR). The NCR contains all cases of cancer in The Netherlands (i.e., total population of 17.4 million), mainly based on notification by the digital pathology archive and the national registry of hospital discharge diagnoses. Independent data-managers collect data on baseline and tumor characteristics as well as treatment and survival data in each Dutch hospital based on hospital records. Full histopathology reports were requested from The Nationwide Network and Registry of Histo- and Cytopathology in The Netherlands (PALGA) [7]. This registry contains histopathology reports from all Dutch pathology laboratories, including all histopathological examined tissues. All histopathology laboratories are connected to PALGA via a special network that enables collection of the histopathology reports. Both NCR and PALGA are independent organizations, funded by the Dutch government. This study is reported in accordance with the STROBE guidelines [8].

### Study population

Patients with histopathologically proven SB-NEN of any stage and differentiation grade were included. The diagnosis was based on the International Classification of Disease-Oncology (ICD-O-3) topography and morphology codes [9]. Surgical approach (open/laparoscopic) is registered in the NCR since 2010, hence only patients with a diagnosis between 2010 and 2015 were included for the present study. Exclusion criteria were: grade 3 NEN, mixed neuroendocrine-non-neuroendocrine tumors (MiNEN), duodenal NENs, double tumors (e.g., concomitant SB-NEN and adenocarcinoma of the colon), autopsy and cytology data, benign neoplasms and non-neuroendocrine neoplasms. Grade 3 NEN were excluded because of the essentially different prognosis and rarity for small bowel localization, therefore it should be considered a separate disease entity.

### Data collection

Primary tumor location was classified as jejunum (C17.1), ileum (C17.2) or small bowel not otherwise specified (C17.9), according to the ICD-O-3 codes. Missing TNM stage was assessed using supplementary data on “extend of disease” present in the NCR database.

Data in both NCR and PALGA databases correspond based on unique NCR-codes. This feature was used to couple both datasets. Data regarding topography, differentiation grade, resection margins, TNM staging and tumor positive lymph nodes were extracted from the full histopathology reports provided by PALGA. Morphology codes were used in case of a mismatch in differentiation grade [10]. Data from PALGA prevailed, in case of disagreement between both datasets. Finally, all tumors were restaged according to the 8th edition of the TNM classification [11].

In case of multiple histopathology reports (e.g., two biopsies followed by a resection), the first date was used as ‘date of diagnosis’. Time to treatment analyses could not be performed because the diagnosis was based on pathology data, which was often the date of surgery. Overall survival was defined as the time between date of diagnosis and date of death or censored at the end of follow-up.

### Statistical analysis

Categorical data are presented as number of cases and percentages, whilst continuous data are presented as either mean with standard deviation (SD) or median with interquartile range (IQR), depending on the data distribution. Overall survival analyses were performed using the Kaplan–Meier method and compared with the Log-Rank test. Univariable and multivariable Cox proportional hazards regression models were used to estimate hazard ratios (HR) with 95% confidence intervals (CI) to identify factors associated with overall survival. Factors with a p value < 0.2 in univariable analyses were added to multivariable analyses in a forward stepwise fashion. The study period was divided into two time periods (2010–2012 and 2013–2015), and added to the Cox proportional hazards regression model to correct for historical improvements in outcomes. A two sided p value ≤ 0.05 was considered statistically significant. Data were analyzed using the Statistical Package for Social Sciences (SPSS) version 26 (IBM Corp. Armonk, NY, USA).

## Results

In total, 482 patients were included over a period of six years (2010–2015), of whom 342 (71%) underwent open and 140 (29%) laparoscopic resection (Table 1). There was a minor increase in the proportion of laparoscopic resections during the study period: 46% (2010–2012) versus 54% (2013–2015). Academic centers performed less often laparoscopic resections than regional hospitals (24/121 (20%) vs. 111/339 (33%), *p* = 0.012). Patients undergoing open resection were more often male (58% vs. 43%, *p* = 0.003) and older (64 vs. 60 years, *p* = 0.009) compared to patients undergoing laparoscopic resection. Emergency procedures constituted a minority of patients, with a slightly skewed distribution toward more emergencies in the open group: 5% versus 3% obstruction (*p* = 0.36) and 2% vs. 0% perforation (*p* = 0.07), respectively.

**Table 1:** Patient and pathology characteristics

	Surgery, no. (%)^a^		
Characteristics, no. (%)	Open (*n* = 342)	Laparoscopic (*n* = 140)	*p* value
Diagnosis year			
2010–2012	169 (49)	65 (46)	0.55
2013–2015	173 (51)	75 (54)	
Treatment center^b^	335	135	
Regional hospital	238 (71)	111 (82)	0.012
Academic center	97 (29)	24 (18)	
Sex			
Male	197 (58)	60 (43)	0.003
Age, years, mean (SD)	64 (12)	60 (12)	0.009
Tumor grade: no evaluable	341	138	
Grade 1	270 (79)	110 (80)	0.90
Grade 2	71 (21)	28 (20)	
Clinical TNM classification^b^			
registered cT stage	88	33	< 0.001
cT4	26 (30)	4 (12)	
registered cN stage	259	109	< 0.001
cN1-2	165 (64)	37 (34)	
registered cM stage	341	139	< 0.001
cM1	140 (41)	28 (20)	
Pathological T classification^b^	320	133	
pT1	8 (2)	20 (15)	< 0.001
pT2	31 (10)	20 (15)	
pT3	169 (53)	56 (42)	
pT4	112 (35)	37 (28)	
Pathological N classification^b^	296	123	
pN0	45 (15)	24 (20)	0.018
pN1	198 (67)	90 (73)	
pN2	53 (18)	9 (7)	
Pathological M classification^b^			
pM1	112 (33)	28 (20)	0.005
Multifocal tumors	81 (24)	19 (14)	0.014
Size of (largest) primary tumor, mm, mean (SD)	21 (10)	18 (10)	0.007
Lymph nodes			
Number of examined LNs, mean (SD)	12 (10)	11 (7)	0.81
Number of tumor positive LNs, mean (SD)	3 (4)	3 (3)	0.43
Disease stage^b^	312	124	
Stage I-II	25 (8)	17 (14)	0.012
Stage III	175 (56)	79 (64)	
Stage IV	112 (36)	28 (22)	
Resection margin	300	118	
R0	244 (81)	105 (89)	0.06
R1/2	56 (19)	13 (11)	
Conversion rate^c^	–	8/22 (36)	–
30-day mortality	16 (5)	3 (2)	0.19

^a^Unless stated otherwise

^b^Data are reported for evaluable cases

^c^Conversion rates were only reported in 2015, during which 22 laparoscopic resections were performed

*SD* Standard deviation, *mm* millimeter, *LN* Lymph node

Patients in the open resection group had a significantly higher clinical stage of NEN with higher proportions of cN1-2 and cM1 stage. Also pathological outcomes were significantly different between the two surgical approaches, with higher pT, pN and pM stages in the open group, as well as a higher percentage of multifocal tumors and larger size of the (largest) primary tumor. A trend toward more positive resection margins in the open resection group was observed (19% vs. 11%, *p* = 0.06).

Conversion rate was only available for the year 2015, in which 8 of 30 (27%) laparoscopic procedures were converted. Although no strict reasons for conversion were documented, the following outcomes were observed: pT4 tumors were present in 5/8 (63%) patients, multifocal tumors in 3/8 (38%), pN2 lymph node metastases in 2/8 (25%) and R1/2 resection margin in 1/8 (13%) patients. Mean (SD) tumor size was 27 (9) in the converted cases and 21 (9) mm in the non-converted cases (*p* = 0.10).

**Fig. 1:**
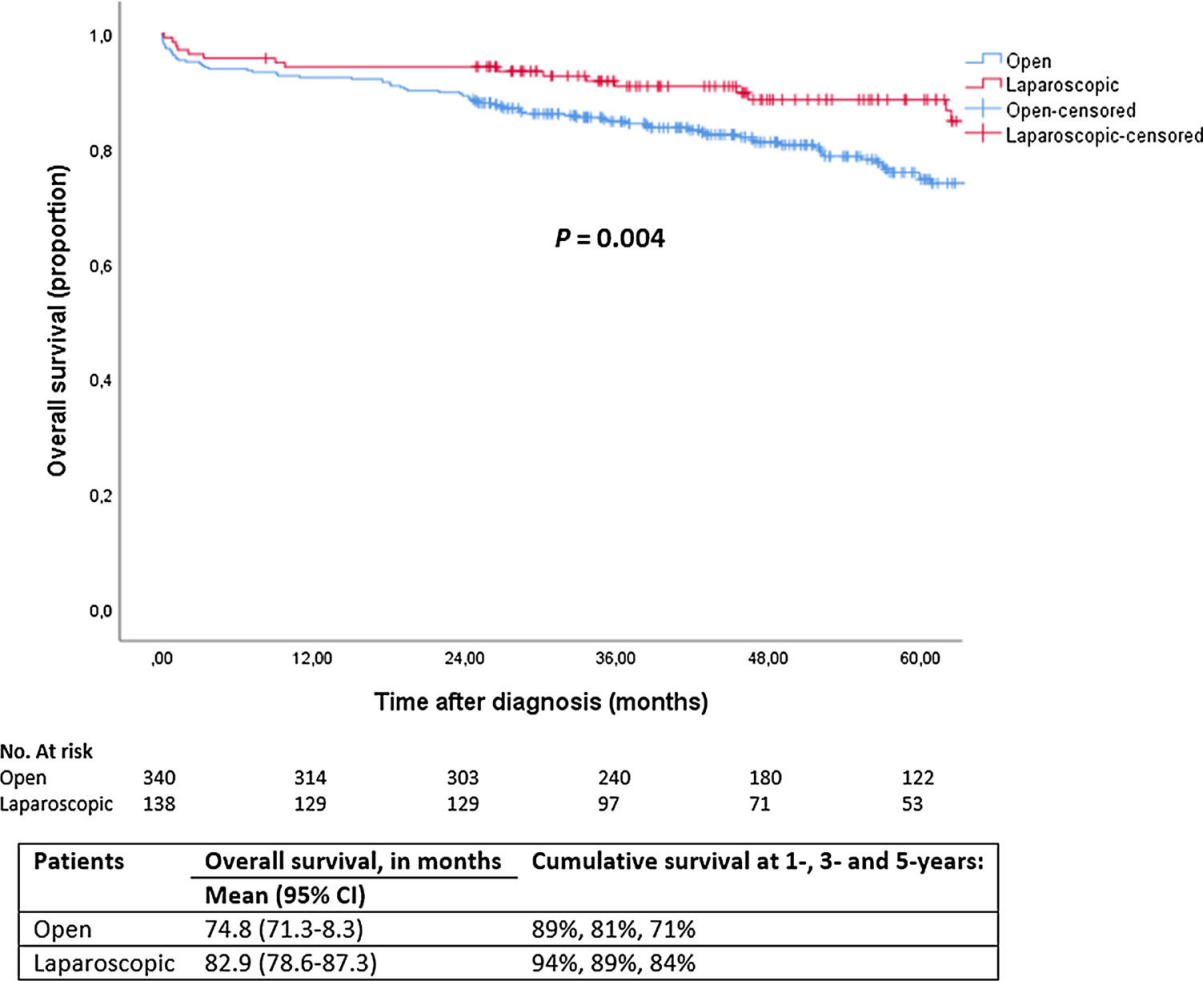
Overall survival, stratified by surgical approach

**Fig. 2:**
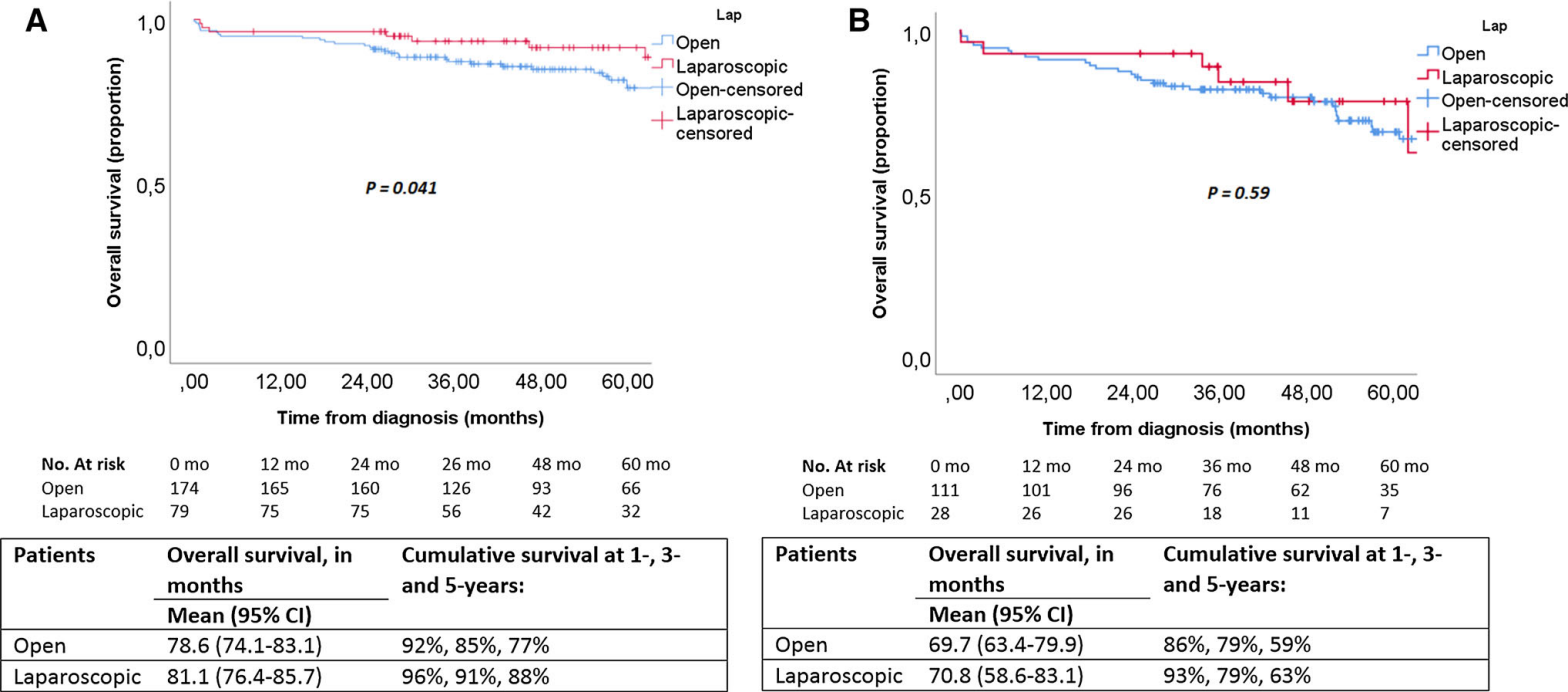
Overall survival for **a** stage III, and **b** stage IV disease, stratified by surgical approach

Within 30 days postoperatively, 16 patients (5%) died in the open group and 3 patients (2%) after laparoscopic resection (*p* = 0.19). Estimated 5-year overall survival of the entire cohort (i.e., patients amenable for resection) was 74%. Without correction for confounders, patients undergoing laparoscopic resection had significantly higher 5-year overall survival rates compared to open resection: 84% versus 71% (*p* = 0.004), respectively (Fig. 1). Survival rates were also separately analyzed for stage III and stage IV disease (Fig. 2). A statistically significant higher 5-year overall survival was found after laparoscopic surgery in stage III patients (88% vs. 77%; *p* = 0.041), while there was no significant difference between the two surgical approaches for stage IV (59% vs. 63%; *p* = 0.59).

In univariable analysis, age above 60 years, multifocal tumors, stage IV disease and laparoscopic resection showed an association with overall survival (Table 2). In multivariable analyses, age between 60 and 75 years (HR 3.39, 95% CI [1.85–6.25], *p* < 0.001) and ≥ 75 years (HR 7.69, 95% CI [3.89–15.18], *p* < 0.001), stage IV disease (HR 1.89, 95% CI [1.20–2.99], *p* = 0.006), and laparoscopic resection (HR 0.52, 95% CI [0.28–0.95], *p* = 0.032) remained significantly associated with overall survival. The results of univariable and multivariable analyses for overall survival are shown in Table 2.

**Table 2:** Uni- and multivariable survival analyses of patients with SB-NEN in The Netherlands

Risk factors	Univariable analysis		Multivariable analysis	
	HR (95% CI)	*p* value	HR (95% CI)	*p* value
Diagnosis year				
2010–2012	1.47 (0.93 − 2.32)	0.10	1.33 (0.82 − 2.15)	0.25
2013–2015	1 [Reference]	1 [Reference]		
Sex				
Male	1 [Reference]		–	
Female	0.84 (0.57 − 1.25)	0.40	–	
Age				
< 60	1 [Reference]			
60–75	3.20 (1.81 − 5.66)	< 0.001	3.38 (1.84 − 6.23)	< 0.001
≥ 75	6.86 (3.68 − 12.82)	< 0.001	7.63 (3.86 − 15.07)	< 0.001
Multifocal tumors				
No	1 [Reference]		1 [Reference]	
Yes	1.40 (0.90 − 2.19)	0.14	1.25 (0.78 − 2.00)	0.35
Disease stage				
Stage I-II	1.60 (0.73 − 2.50)	0.24	–	
Stage III	1 [Reference]		1 [Reference]	
Stage IV	1.96 (1.25 − 3.06)	0.003	1.86 (1.18 − 2.94)	0.043
Tumor grade				
Grade 1	1 [Reference]		–	
Grade 2	1.33 (0.83 − 2.14)	0.24	–	
Resection margin				
R0	1 [Reference]		–	
R1/2	1.16 (0.67 − 1.99)	0.60	–	
Surgery				
Open	1 [Reference]	1 [Reference]		
Laparoscopic	0.47 (0.28 − 0.80)	0.005	0.51 (0.28 − 0.94)	0.032

## Discussion

The main finding of this nationwide study was that 29% of the patients with SB-NEN were planned for a laparoscopic approach. There was a slight but non-significant increase in laparoscopic resection rate over time. Case selection was clearly seen, with less favorable tumors in patients who underwent open resection, as reflected by significantly higher stage, larger size, and more multifocal tumors. Academic centers performed less laparoscopic resections as compared to regional hospitals, likely reflecting tertiary referral of more advanced cases. With the available variables in the dataset, the association between surgical approach and overall survival was corrected for confounding as much as possible. The multivariable model revealed better overall survival after a laparoscopic approach, with age and stage as the other independent predictors. Potential prognostic factors such as margin status, grade and multifocal tumor location were not found to be associated with overall survival in this patient cohort.

The application of a laparoscopic approach for resection of SB-NEN is mainly determined by the extensiveness of mesenteric lymph node metastases. Ohrvall et al. proposed a classification of these metastases, ranging from resectable stage I (close to the intestine) to irresectable stage IV (retroperitoneal, peri-pancreatic or encasement of the mesenteric artery with involvement of proximal jejunal arteries) [12]. In this study, 38% of lymph node metastases extended along the superior mesenteric artery without encasement, whilst 16% were irresectable. Depending on the laparoscopic experience, one would expect that at least 40% of patients are amenable to a laparoscopic approach based on these data. The 29% laparoscopic resection rate as found in the present study suggests a still restricted application.

The recent European Society of Medical Oncology guideline states that patients with SB-NEN often present in the emergency setting [13]. An emergency resection without prior knowledge of the presence or nature of a small bowel neoplasm might lead to oncologic inferior resections. Interestingly, emergency resections for obstruction or perforation were performed in only a small minority (8%) of this nationwide cohort. This finding suggests that patients might have been offered ‘up-front’ resection to prevent bowel obstruction and/or ischemia [13]. However, the value of ‘up-front’ resection for SB-NEN has been debated in literature, as this is associated with significantly more reoperations, rather than yielding a survival advantage [14].

A common misunderstanding of laparoscopic resection is the inability of palpating the small bowel. However, after completion of the lymph node dissection, almost the entire small bowel can be exteriorized through an umbilical extraction site, enabling meticulous palpation [15]. Identification of multifocal primary disease is regarded a critical step during resection, although recent analyses suggest that the presence of multifocal disease does not affect overall survival [5, 16]. Therefore, we carefully hypothesize that multifocality is not a contraindication for laparoscopic resection. Nevertheless, multifocal primary tumors were more often found in the open resection group, although there might not be a causal relationship, but rather a reflection of case selection and more advanced tumor stage.

The conversion rate (36%) was only reported in the last registration year (2015) and was higher compared to previous studies (25–30%) [15, 17]. In contrast, the conversion rate for laparoscopic D3 lymphadenectomy for colon cancer was 5% in a randomized clinical trial [18]. The substantially higher conversion rate reflects the level of complexity that might be encountered during resections for SB-NEN. The essential difference between central mesenteric lymph node metastases that originate from colon cancer or SB-NEN is related to the infiltrative growth with sometimes extensive mesenteric fibrosis and vascular encasement in the latter tumor type. Likely, D3 lymphadenectomy requires even more skills if performed for SB-NEN than for colon cancer. A handport-assisted laparoscopic procedure can sometimes be an alternative for conversion.

Pedrazzani et al. described a case series of nine patients undergoing laparoscopic right hemicolectomy with complete mesocolic excision for terminal ileum/right colon/appendix NEN [19]. Although it comprises a small cohort, peri-operative and long-term survival outcomes were promising: 1/9 had a Clavien-Dindo grade III complication, no mesenteric locoregional recurrence and all patients with an R0 resection were disease free after a median follow-up of 18 months (range 6–50). One randomized trial of laparoscopic versus open D3 lymphadenectomy has been published in stage II-III colon cancer, which revealed beneficial short-term outcomes for minimally invasive surgery (less blood loss, shorter time to pass first flatus, decreased use of postoperative analgesics and shorter hospital stay), without compromising 5-year overall survival [18, 20]. Interestingly, a recent meta-analyses that pooled the results of this trial with comparative cohort series, suggested that laparoscopic resection was even associated with better oncological outcomes for colon cancer [21], similar to the present study. However, the methodological issues of non-randomized comparisons do not allow for definitive conclusions on this observed association. There is a high risk of bias, and laparoscopic resection might just be a reflection of treatment by more specialized surgeons in dedicated centers with optimized peri-operative care.

Long-term nationwide population-based data were used for this study, making it more representative than cohort studies. However, the findings of this study should be seen in light of some limitations. The NCR database is primarily focused on oncologic characteristics, which limits analysis of (peri-) operative characteristics (indication, conversion rate, postoperative morbidity) and imaging data such as postoperative CT or PET scanning to assess the completeness of mesenteric lymphadenectomy. Time to recurrence, which is a relevant marker of “surgical success” in regard to lymphadenectomies, could not be reported, as recurrent disease is often not diagnosed with biopsies. The most important missing data concern the reasons for choosing an open or laparoscopic approach and whether surgery was performed in the emergency setting.

We propose that guidelines should adapt their recommendations regarding selection criteria for laparoscopic resection, for example using the classification as proposed by Ohrvall et al. [12]. It seems that more SB-NENs are eligible for laparoscopic resection, especially in hands of colorectal surgeons with experience in D3 lymphadenectomies. Also, less emphasis on multifocality as a reasons not to perform laparoscopic resection should be given.

Further work is required to establish the role of laparoscopic resection for SB-NEN. Ideally, this would be a multicenter international randomized clinical trial with stratification for extent of lymph node metastases. However, such a trial would be challenging because of the rarity of the disease and potential lack of equipoise. To overcome this issue, a prospective international cohort study with clearly documented inter-hospital variability in surgical practice could also provide relevant data. To really be of added value, such a study should include variables that reflect “surgical success”, which are part of standard follow-up protocols (pathology reports of recurrent lesions, blood tests, PET/CT) [3].

In conclusion, this study showed that laparoscopic resection of SB-NENs was performed in 29% of patients in the Netherlands. Current data do not raise major concern regarding oncologic adequacy of laparoscopic resection in selected cases.
